# Patients in mental healthcare should be referred to as patients and not service users

**DOI:** 10.1192/bjb.2021.40

**Published:** 2021-12

**Authors:** Stefan Priebe

**Affiliations:** Unit for Social and Community Psychiatry, Queen Mary University of London, UK

**Keywords:** Mental disorders, health services, patients, service users, patient preference

## Abstract

Over the past few years the term ‘service users’ has been increasingly used to describe patients in mental healthcare. This paper argues that the term ‘service user’ in this context should be avoided and outlines four reasons: the term is discriminating, cynical, patronising and detrimental. Of course, none of these effects is intentional, but that does not change them. The term ‘patient’, however, describes appropriately a temporary role in healthcare, provides parity of esteem with patients in physical healthcare and reflects the reasons why large parts of society are willing to fund healthcare, in solidarity with those who are sick.

The terms with which we name similar objects and roles can change over time. One reason may be that a term is seen as devaluing or linked with connotations that one would like to change. Psychiatry has a long history of examples of this. Terms such as ‘madness’ have been replaced by more medical terms such as ‘mental disorders’ to emphasise that one is dealing with a health problem. Another example is seen in the former asylums, which tended to change their names to avoid the negative connotations associated with a previous name that had been built up in the population over time. For example, the Karl-Bonhoeffer-Nervenklinik (last of the changed names) in Berlin changed its name four times within a period of only 100 years.^1^

Recently, there has been a shift in the National Health Service (NHS) towards calling patients in mental healthcare ‘service users’ instead of patients. The term is used in guidelines published by the National Institute for Health and Care Excellence (NICE),^2^ in publications of voluntary organisations^3^ and in prestigious scientific journals.^4^ Of course, every individual should be entitled to be addressed in any way they like, but the question is whether the term service user should be generally used when referring to patients.

I will argue that the term service user should be avoided, for four reasons: because it is discriminating, cynical, patronising and detrimental. Of course, these effects are not intentional, but that does not prevent their ultimate harm.

## Discriminating

The term patient describes a temporary and context-dependent role. When I see my general practitioner (GP) in their clinic, I am a patient. When I see the same person in a different context, we have different roles. I may be a neighbour, a fellow passenger on the same bus, a father of a child that goes to school with their child, or the GP may even be a patient in my clinic. There are endless possibilities. However, the term patient is a precise description of a temporary role in a professional health service, without any negative connotation. Health services have patients, whereas lawyers, insurance companies and restaurants have clients or customers or consumers, but never patients. The term patient applies to all types of health service and has only rarely been challenged outside mental health. If mental health services now diverge from other health services and decide to call their patients by another term, they turn their patients into something different. Parity of esteem – defined as ‘valuing mental health equally with physical health’^5^ – is undermined when mental health services use terms that distinguish their patients from patients in other services and, thus, discriminate against their own patients.

## Cynical

Service user, as a term, suggests that the people in question either ‘use’ the services actively or that the service has been of ‘use’ to them. Neither of these assumptions necessarily applies. In England alone, more than 50 000 times a year patients are treated involuntarily, i.e. against their wishes and involving specific legislation that allows such coercion.^6^ One can hardly claim that those people ‘use’ the service, just as prisoners are not ‘prison users’. Also, although mental healthcare is hopefully beneficial to many patients, it would be grandiose of professionals and others to believe that mental healthcare helps everybody. Thus, it is not universally of ‘use’ and there is no question that in some cases – despite the best intentions of all people involved – it might even be harmful. Thus, both suggestions of the meaning of ‘use’ that are inherent in the term service user may be regarded as cynical.

## Patronising

A number of surveys have asked patients in mental health services which term they prefer to be used. The results of these surveys are consistent. The majority of patients prefer the term patient, and this applies across studies that have been conducted at different times and in different settings.^7–9^ Insisting on a term that most of the patients explicitly do not want may be seen as patronising.

## Detrimental

This may be the most complicated of the four points. Healthcare – at least in most European countries – is paid for through the solidarity of the population, either by sharing contributions and benefits through health insurances or in tax-funded healthcare systems. With respect to the NHS in the UK, most people in the population have little problem with paying their taxes so that people who suffer from illnesses can receive proper healthcare when they need it. This may be motivated by the expectation that each taxpayer will also receive tax-funded care when they need it, but is also based on cultural values (e.g. of Christianity and Enlightenment) and the compassion for those who suffer. As a society we accept that some people are sick and need professional – and potentially expensive – treatment for as long as they are sick. This is reflected in the term patient (originating from the Greek ‘pathos’ and Latin *patiens*, which denote suffering). When mental healthcare providers expect the population to fund their work, then the term service user is not helpful. It rather evokes the idea of a ‘service’ that someone decides to use or not to use, instead of the professional care that some people receive because they are so seriously distressed that they need that care.

## Conclusions

I would therefore argue that the term patient should be re-established in mental healthcare in the NHS. In this brief paper, the argument focused on the alternative term service user. Similar arguments could be made about other terms, such as ‘client’ and ‘consumer’. Those arguments would overlap in parts with the ones put forward here (see ref.^9^).

I have personally experienced the strong views and feelings of present and former patients in NHS mental health services who prefer the term service user. As noted at the beginning, such views should be respected, as long as the requested terminology applies to those people themselves and not to everybody else. However, the general terminology that is used in mental health services should not be determined by the specific views of a minority of patients and/or professionals. Mental healthcare is based on shared values and scientific evidence. Both require precise thinking, and precise thinking requires an exact and consistent terminology.

## About the author

**Stefan Priebe**, Dipl Psych, Dr. med. habil., is Professor of Social and Community Psychiatry in the Unit for Social and Community Psychiatry at Queen Mary University of London. The unit is a World Health Organization (WHO) Collaborating Centre for Mental Health Service Development, based at the Newham Centre for Mental Health, London, UK.
